# Supplementary material for: Sleep spindle architecture associated with distinct clinical phenotypes in older adults at risk for dementia
Source: Mol Psychiatry. 2023 Dec 5;29(2):402–11. doi: 10.1038/s41380-023-02335-1 (PMC11116104; doi:10.1038/s41380-023-02335-1)
Supplement: Supplementary file 1 — Supplementary Material [file 41380_2023_2335_MOESM1_ESM.docx]

**Supplementary Material:** Sleep spindle architecture associated with distinct clinical phenotypes in older adults at risk for dementia

**1.** Automated spindle detection algorithm validation Page 2

**2.** Resting state fMRI pre-processing Page 5

**3.** Cluster identification consensus-based algorithm results Page 6

**4.** Sensitivity analysis for PSG system effect Page 7

**5.** Additional neuropsychological and sleep micro- and macro - architecture measures Page 10

**6.** Resting state fMRI supplementary analysis Page 12

**1. Automated spindle detection algorithm validation**

Please note, the following section is taken from the supplementary material reported by our team previously (1).

*Reference standard: Manual Spindle Identification*

Sleep spindles from 200 epochs per study of stage N2 sleep were manually identified at the central EEG derivation referenced to the right mastoid electrode (C3-M2) in a sample of 10 (5 Mild Cognitive Impairment (3 male, mean age 70.8 ± 6.0 years); 5 Healthy Older Adults (4 male, mean age 68.0 ± 9.1 years)) all night in-laboratory polysomnography recordings by trained sleep technologists.

Studies were visually inspected in 30-second epochs using REMLogic software (Natus, San Carlos, CA, USA), by plotting both raw EEG (C3-M2) trace and a filtered trace (11-26.5Hz) on the display. The criteria for manual spindle identification involved: a clear, distinct spindle event observable in the raw trace; with frequency range of 11-16Hz; a duration of 0.3-3 seconds and comprising a diamond shape. In the case of partially overlapping spindles or a short inter-spindle distance, two separate spindles were scored if the sigma activities returned to baseline amplitude and duration criteria were satisfied. Reference standard spindle events were marked from onset to offset and exported with start time and duration details.

*Automated Sleep Spindle Detection Algorithm*

An automatic sleep spindle detection tool was developed and written in Java, version 1.6 (Oracle, Santa Clara, CA, USA). The tool’s algorithm computationally performs the following steps which are depicted in *Supplementary Figure S1*. First, a 128 order band-passing Finite-Impulse-Response filter (11-16 Hz) is applied to the raw EEG signal, yielding a time course of sigma activity with duration threshold 0.5 ≤ duration ≤ 3.0 seconds. A Hilbert transformation is then applied to extract envelopes of the sigma activities. The spindles were identified according to the relative amplitude threshold, where the relative threshold value was calculated by the formula: median + α * standard deviation of the amplitude (µV) and calculated independently for each EEG derivation. For the relative amplitude method, the performance of the algorithm was tested against the reference standard at multiple α values in range 0.8 ≤ α ≥ 2.0. In the case of partially overlapping spindles or a short inter-spindle distance, spindles were considered a single event when the inter-spindle amplitude was above threshold and duration criteria was satisfied. The spindle algorithm was applied to a central EEG signal (C3-M2) to identify spindles in 200 epochs of stage N2 sleep corresponding to the same epochs used in the reference standard.

**Supplementary Figure 1**

**Automatic algorithm spindle detection schematic.** Five second epoch of artefact free EEG. Raw, raw EEG signal; Filtered, signal filtered for sigma activity; Envelope, envelope of activity in the frequency range of sigma and meeting duration criteria.

Accuracy was evaluated using an F_1_-score to compare automated and manual (gold standard reference) methods of spindle detection. F-1 scores range between 1 and 0, where 1 is equivalent to perfect precision and recall. Finally, the F_1_-score generated from our automated spindle detection algorithm was compared to six published algorithms that were previously evaluated (2).

*Algorithm Performance*

### Supplementary Table 1 shows the F_1_-scores for the automated spindle detection algorithm used in the current study and other published algorithms. The optimal α amplitude threshold value of 1 was chosen based on previous training and validation of the algorithm in middle-aged OSA and healthy adults, and based on this threshold the F_1_-scores of the algorithm were 0.51 and 0.49 for Healthy Older Adults and Mild Cognitive Impairment groups, respectively. When compared to the previously published algorithms (Warby et al. 2014), our algorithm demonstrated similar F_1_-scores to two of the highest scoring algorithms and were higher than the F_1_-scores of four other algorithms.

**Supplementary Table 1**

| **Performance of different spindle detection algorithms** | | **F1-scores** |
| --- | --- | --- |
| *Automated Spindle Detection Algorithm used in current study* | | |
|  | Healthy Older Adults | 0.51 |
|  | Mild Cognitive Impairment | 0.49 |
| *Spindle Detection Algorithms detailed by Warby et al. 2014* | | |
|  | Algorithm 1 | 0.28 |
|  | Algorithm 2 | 0.28 |
|  | Algorithm 3 | 0.21 |
|  | Algorithm 4 | 0.50 |
|  | Algorithm 5 | 0.52 |
|  | Algorithm 6 | 0.41 |
| F_1_-scores for Algorithm 1-6 are from Warby et al. 2014. | | |

**2. Resting state fMRI pre-processing**

Pre-processing was performed in MATLAB using the functional connectivity toolbox (CONN v19.c; <http://www.nitrc.org/projects/conn>).

Pre-processing involved a standardised Montreal Neurological Institute (MNI)-space direct normalisation pipeline (described in detail here (3). Briefly, each volume was co-registered and resampled to a reference volume (i.e., the first scan) using b-spline interpolation. Rigid head movement timeseries were calculated (6 degree of freedom) and added as a first-level covariate for motion correction (see aCompCor below). Slice-timing correction using sinc-interpolation to match the mid-TR time was then performed. Outlier scans were identified and defined as volumes with frame-wise displacement greater than 0.5mm or signal intensity changes greater than three standard deviations. Functional and structural data were the normalised to MNI standard space and segmented into grey matter, white matter, and cerebrospinal fluid using the SPM12 unified segmentation and normalisation procedure, which includes estimating the best non-linear spatial transformation (4). This procedure was applied to the functional data using the mean blood-oxygen-level-dependent signal as the reference image, and to the structural data using the raw T1-weighted volume as the reference image. Data were resampled to 2 mm isotropic voxels for functional data and 1 mm isotropic voxels for structural data, using 4th order spline interpolation.

A standard denoising pipeline was applied, involving two steps. First, aCompCor was applied to the BOLD timeseries to regress out noise and motion artefacts. Noise regressors were obtained from white matter and cerebrospinal fluid timeseries, as well as the first-level covariates previously defined (12 components from the estimated subject- motion parameters, derived from three translation and three rotation parameters and their first- order derivatives, and the outlier volumes to be ‘scrubbed’). Second, a temporal band pass filter was applied to the BOLD signal (0.009 – 0.08 Hz) after confound regression to minimise the influence of physiological, head-motion and other noise sources. This was performed after regression to avoid any frequency mismatch in the nuisance regression procedure (5). Images were then visually inspected for quality. Four participants were excluded from further analysis due to poor quality data or artefacts.

**3. Cluster identification consensus-based algorithm results**

An optimal solution of 3 clusters was identified by the consensus-based algorithm. The choice of 3 clusters is supported by 10 (34.48%) methods out of 29 (Ch, Hartigan, Scott, trcovw, Tracew, Ratkowsky, Ball, SDindex, Mixture (VVI), Mixture (EVI)).

**Supplementary Figure 2**

***Optimal number of clusters: method agreement procedure****.*

**4. Sensitivity analysis for PSG system effect**

Here we conduct exploratory analysis of the different systems used during polysomnography (PSG) recording in this study. As the data presented in this study was collected across a period of 10 years (between 2010-2020) and spanned over two sites different sites (Brain and Mind Centre, Sydney, Australia and Woolcock Institute of Medical Research in Sydney, Australia), five different PSG recording systems were used, shown in Table 2.

To confirm there were no systematic differences across the PSG system we conducted a sensitivity analysis. A sensitivity analysis assesses the robustness’ of an analysis by examining the extent to which results are affected by underlying changes in the study design.

**Supplementary Table 2**

| **Code** | **PSG system** | **Sampling rate** |
| --- | --- | --- |
| 0 | Grael | 256 Hz |
| 2 | Alice | 200 Hz |
| 3 | REMLogic | 512 Hz |
| 4 | Sandman | 128 Hz |
| 5 | Siesta | 256 Hz |

***Coded values for PSG system.***

To check that the distribution of PSG system was not skewed across the clusters we performed chi-square and fisher exact tests. We then perform our multinominal logistic regression analysis with and without adjusting for PSG system to test its effect on our measures of interest.

**Supplementary Figure 3**

**Bar chart of PSG system count for each cluster.** Coded values for system outlined in Table 2.

A Pearson’s chi-square test of independence was performed to evaluate the relationship between cluster and PSG system. The relationship between these variables was not significant (χ^2^ (8) = 14.288, *p* = 0.075). Fisher’s exact test was also used to determine if there was a significant association between cluster and PSG system. There was not a statistically significant association between these variables (p = 0.070).

**Supplementary Table 3**

|  |  | **β** | **Odds ratio** | **95% CI** | ***p*-value** |
| --- | --- | --- | --- | --- | --- |
| **Cluster 2^*^** | Memory | -1.22 | 0.30 | 0.13-0.68 | **0.004** |
|  | Sleep quality | 0.01 | 1.01 | 1.00-1.02 | 0.157 |
|  | Oxygen desaturation index | -0.01 | 1.00 | 0.95-1.05 | 0.839 |
| **Cluster 3^*^** | Memory | -1.74 | 0.18 | 0.06-0.51 | **0.001** |
|  | Sleep quality | -0.02 | 0.99 | 0.96-1.01 | 0.224 |
|  | Oxygen desaturation index | 0.07 | 1.07 | 1.01-1.13 | **0.018** |
|  |  |  |  |  |  |

***Model outcomes without adjusting for PSG system.*** *Results of multinomial regression model, with age and sex included as fixed effects (Table from Results). Memory refers to the composite score. Sleep quality measured by WASO. CI = confidence interval. Oxygen desaturation index measured by 3% ODI events per hour.*

**Supplementary Table 4**

|  |  | **β** | **Odds ratio** | **95% CI** | ***p*-value** |
| --- | --- | --- | --- | --- | --- |
| **Cluster 2^*^** | Memory | -1.31 | 0.27 | 0.11-0.69 | **0.006** |
|  | Sleep quality | 0.01 | 1.01 | 0.99-1.03 | 0.226 |
|  | Oxygen desaturation index | 0.002 | 1.00 | 0.95-1.06 | 0.953 |
| **Cluster 3^*^** | Memory | -1.90 | 0.15 | 0.05-0.50 | **0.002** |
|  | Sleep quality | -0.02 | 0.98 | 0.95-1.00 | 0.148 |
|  | Oxygen desaturation index | 0.09 | 1.09 | 1.00-1.16 | **0.006** |
|  |  |  |  |  |  |

***Model outcomes adjusting for PSG system.*** *Results of multinomial regression model, with age, sex and PSG system included as fixed effects. Memory refers to the composite score. Sleep quality measured by WASO. CI = confidence interval. Oxygen desaturation index measured by 3% ODI events per hour.*

**5. Additional neuropsychological and sleep macro- and micro-architecture measures**

**Supplementary Table 5**

|  |  | **Group 1** | **Group 2** | **Group 3** | ***p* value** | **Pairwise** |
| --- | --- | --- | --- | --- | --- | --- |
| Learning | Logical Memory I, raw score | 42.07 (8.89) | 30.47 (12.44) | 33.22 (13.18) | **< .001** | *1 vs 2; 1 vs 3* |
|  | RAVLT 1-5, raw score | 51.29 (8.69) | 39.23 (12.73) | 43.61 (13.09) | **< .001** | *1 vs 2* |
| Executive function | FAS, total words | 42.41 (13.20) | 37.21 (9.99) | 44.11 (15.53) | .078 | - |
|  | Digit Span, total | 16.58 (3.81) | 16.46 (3.71) | 17.00 (4.77) | .890 | - |
|  | Trailmaking Part B, secs | 79.24 (30.56) | 99.82 (38.74) | 84.91 (33.40) | .051 | - |
|  |  |  |  |  |  |  |

***Additional neuropsychological characteristics of k-means defined cluster groups.*** *Data presented as mean (standard deviation). Group differences compared using one-way analysis of variance with post hoc Tukey’s honestly significant difference. P-values shown for group level comparisons, with significant (p < .05) pairwise comparisons noted. Learning was assessed via i) the sum of the five learning trials the Rey Auditory Verbal Learning Test (RAVLT 1-5; (6)); ii) the encoding part of the Logical Memory subtest of the Wechsler Memory Scale - III (7). Executive function was assessed via: i) Controlled Oral Word Association Test, phonemic fluency (FAS; (8), with the total amount of words generated across three 1-minute periods the outcome of interest; ii) working memory maintenance and manipulation, as measured by score from the forwards and backwards digit span task (Digit Span; (7)); iii) attentional set shifting, measured by the Trail Making Test Part B (Trails B; (9)).*

**Supplementary Table 6**

| **Macroarchitecture** | **Cluster 1** | **Cluster 2** | **Cluster 3** | ***p* value** | **Pairwise** |
| --- | --- | --- | --- | --- | --- |
| Total time in bed (min) | 427.63 (51.14) | 461.46 (58.23) | 421.40 (46.29) | **0.010** | *1 vs 2; 2 vs 3* |
| Total sleep time (min) | 341.60 (54.7) | 346.3 (75.3) | 349.2 (55.9) | 0.920 | - |
| Stage N1 sleep (min) | 20.20 (16.77) | 24.85 (19.78) | 17.87 (10.62) | 0.300 | - |
| Stage N2 sleep (min) | 185.00 (42.67) | 161.90 (46.73) | 198.20 (57.36) | **0.018** | *2 vs 3* |
| Stage N3 sleep (min) | 75.98 (27.04) | 84.69 (48.08) | 76.78 (36.86) | 0.620 | - |
| REM sleep (min) | 60.40 (21.22) | 70.73 (30.32) | 52.55 (28.89) | 0.052 | *-* |
| Sleep efficiency (%) | 80.22 (11.54) | 74.80 (13.46) | 83.07 (11.03) | **0.044** | *2 vs 3* |
| Sleep latency (min) | 23.15 (25.72) | 23.37 (25.13) | 25.54 (28.87) | 0.950 | - |
| REM latency (min) | 136.60 (81.37) | 108.8 (63.17) | 137.90 (86.12) | 0.220 | - |
| AHI (events/hour) | 13.52 (13.86) | 14.65 (14.29) | 24.97 (19.94) | **0.041** | - |

***Sleep macroarchitecture characteristics of k-means defined cluster groups.*** *Data presented as mean (standard deviation). Group differences compared using one-way analysis of variance with post hoc Tukey’s honestly significant difference. P-values shown for group level comparisons, with significant (p < .05) pairwise comparisons noted. Sleep efficiency calculated as ratio of total sleep time to time in bed, multiplied by 100 to display as a percentage NREM min calculated as sum of N2 min and N3 min. AHI; Apnea hypopnea index calculated as the number of apneas and hypopneas per hour of total sleep time.*

**Supplementary Table 7**

| **Spindle architecture** | **Cluster 1** | **Cluster 2** | **Cluster 3** |
| --- | --- | --- | --- |
| NREM fast spindle density (events per min) | 1.10 (0.55) | 0.21 (0.26) | 0.30 (0.24) |
| NREM spindle amplitude (µV) | 15.87 (3.15) | 13.84 (3.25) | 24.81 (5.54) |
| NREM spindle duration (seconds) | 0.81 (0.03) | 0.72 (0.03) | 0.75 (0.04) |

***Sleep spindle architecture of k-means defined cluster groups.*** *Data presented as mean (standard deviation).*

**6. Resting state fMRI supplementary analysis and figures**

**Supplementary Table 8**

| **Spindle measure** | **Network** | **Main effect (connectivity)** | **Interaction effect (connectivity: cluster)** |
| --- | --- | --- | --- |
| Fast density | CO | F(1,76) = 0.36, *p* = 0.552 | F(1,76) = 0.40, *p* = 0.671 |
|  | DAN | F(1,76) = 0.13, *p* = 0.724 | F(1,76) = 0.02, *p* = 0.985 |
|  | FPN | F(1,76) = 0.48, *p* = 0.492 | F(1,76) = 0.06, *p* = 0.940 |
|  | RSP | F(1,76) = 0.90, *p* = 0.347 | F(1,76) = 1.33, *p* = 0.271 |
|  | VAN | F(1,76) = 1.64, *p* = 0.204 | F(1,76) = 0.78, *p* = 0.463 |
| Duration | CO | F(1,76) = 2.47, *p* = 0.120 | F(1,76) = 1.15, *p* = 0.323 |
|  | DAN | F(1,76) = 3.06, *p* = 0.084 | F(1,76) = 0.88, *p* = 0.419 |
|  | FPN | F(1,76) = 0.02, *p* = 0.898 | F(1,76) = 0.56, *p* = 0.574 |
|  | RSP | F(1,76) = 2.04, *p* = 0.158 | F(1,76) = 1.30, *p* = 0.279 |
|  | VAN | F(1,76) = 0.85, *p* = 0.359 | F(1,76) = 1.60, *p* = 0.208 |

***Output from additional functional connectivity linear models.*** *Models examining main effect of functional connectivity and interaction effect of functional connectivity and cluster membership on sleep spindle characteristics. Amplitude not investigated as no significant effect of default mode connectivity emerged. CO = cingulo opercular, DMN = dorsal mode network, DAN = dorsal attention network, FPN = frontoparietal network, RSP = Retrosplenial network VAN = ventral attention network. Salience network excluded from further analysis due to small size (number of regions = 5).*

**References**

1. Lam A, Haroutonian C, Grummitt L, Ireland C, Grunstein RR, Duffy S, et al. Sleep-Dependent Memory in Older People With and Without MCI: The Relevance of Sleep Microarchitecture, OSA, Hippocampal Subfields, and Episodic Memory. Cerebral Cortex. 2021 Jun 1;31(6):2993–3005.

2. Warby SC, Wendt SL, Welinder P, Munk EGS, Carrillo O, Sorensen HBD, et al. Sleep-spindle detection: crowdsourcing and evaluating performance of experts, non-experts and automated methods. Nat Methods. 2014 Apr;11(4):385–92.

3. Nieto-Castanon A. Handbook of functional connectivity Magnetic Resonance Imaging methods in CONN. Hilbert Press; 2020.

4. Ashburner J, Friston KJ. Unified segmentation. Neuroimage. 2005;26(3):839–51.

5. Hallquist MN, Hwang K, Luna B. The nuisance of nuisance regression: spectral misspecification in a common approach to resting-state fMRI preprocessing reintroduces noise and obscures functional connectivity. Neuroimage. 2013;82:208–25.

6. Strauss E, Sherman EM, Spreen O. A Compendium of Neuropsychological Tests: Administration, Norms, and Commentary. Oxford University Press; 2006.

7. Wechsler D. WMS-III: Wechsler memory scale administration and scoring manual. Psychological Corporation; 1997.

8. Delis DC, Kaplan E, Kramer JH. Delis-Kaplan executive function system (D-KEFS). Psychological Corporation; 2001.

9. Bowie CR, Harvey PD. Administration and interpretation of the Trail Making Test. Nature protocols. 2006;1(5):2277–81.
